# Fatty-acid binding protein 4 gene variants and childhood obesity: potential implications for insulin sensitivity and CRP levels

**DOI:** 10.1186/1476-511X-9-18

**Published:** 2010-02-15

**Authors:** Abdelnaby Khalyfa, Bharat Bhushan, Mohamed Hegazi, Jinkwan Kim, Leila Kheirandish-Gozal, Rakesh Bhattacharjee, Oscar Sans Capdevila, David Gozal

**Affiliations:** 1 Department of Pediatrics, Comer Children's Hospital, The University of Chicago, Chicago, IL, USA; 2 Division of Sleep and Respiratory Medicine, Department of Pediatrics, The Hospital for Sick Children, University of Toronto, Toronto, ON, Canada; 3 Pediatric Sleep Unit, Division of Neurophysiology, Department of Neurology, Hospital Sant Joan de Déu, Barcelona, Spain

## Abstract

**Introduction:**

Obesity increases the risk for insulin resistance and metabolic syndrome in both adults and children. FABP4 is a member of the intracellular lipid-binding protein family that is predominantly expressed in adipose tissue, and plays an important role in maintaining glucose and lipid homeostasis. The purpose of this study was to measure FABP4 plasma levels, assess FABP4 allelic variants, and explore potential associations with fasting glucose and insulin levels in young school-age children with and without obesity.

**Methods:**

A total of 309 consecutive children ages 5-7 years were recruited. Children were divided based on BMI z score into Obese (OB; BMI z score >1.65) and non-obese (NOB). Fasting plasma glucose, lipids, insulin, hsCRP, and FABP4 levels were measured. HOMA was used as correlate of insulin sensitivity. Four SNPs of the human FABP4 gene (rs1051231, rs2303519, rs16909233 and rs1054135), corresponding to several critical regions of the encoding FABP4 gene sequence were genotyped.

**Results:**

Compared to NOB, circulating FABP4 levels were increased in OB, as were LDL, hsCRP and HOMA. FABP4 levels correlated with BMI, and also contributed to the variance of HOMA and hsCRP, but not serum lipids. The frequency of rs1054135 allelic variant was increased in OB, and was associated with increased FABP4 levels, while the presence of rs16909233 variant allele, although similar in OB and NOB, was associated with increased HOMA values.

**Conclusions:**

Childhood obesity is associated with higher FABP4 levels that may promote cardiometabolic risk. The presence of selective SNPs in the FABP4 gene may account for increased risk for insulin resistance or systemic inflammation in the context of obesity.

## Introduction

Childhood obesity is a serious public health problem that has reached epidemic proportions all over the world [1-3]. Metabolic and cardiovascular complications of obesity in childhood, while less common than in adulthood, may nevertheless include hyperlipidemia, insulin resistance and type 2 diabetes, and systemic low grade inflammation.

In children, the presence of obesity has been associated with increased levels of high sensitivity CRP (hsCRP) [4], as well as other inflammatory mediators [5-9], all of which promote the development of endothelial and metabolic dysfunction [10-14]. However, substantial variability is found among obese children as to the presence of metabolic dysfunction or increased inflammatory markers, suggesting that individual genomic variance may account for such discrepancies.

Fatty acid binding proteins (FABP) are a group of related molecules that serve as intracellular chaperones for lipid moieties, coordinate cellular lipid responses, and thereby play a critical role in metabolic and inflammatory pathways [15,16]. Adipocyte FABP, also known as FABP4, A-FABP, or aP2, was initially detected in mature adipocytes, and plays critical roles in hyperlipidemia, atherogenesis and type 2 diabetes, particularly when obesity is concurrently present [17,18]). FABP4 is produced and released to the circulation, and FABP4 levels appear to correlate with the degree of metabolic dysfunction [19,20]. Similar to adults, children who are obese are more likely to display elevations in FABP4 plasma levels, which will be reduced by interventions leading to weight loss [21,22].

We hypothesized that the presence of obesity in community based children will be associated with increased FABP4 levels which may account for individual discrepancies in the presence or absence of insulin resistance or high sensitivity C-reactive protein (hsCRP) plasma levels, and that single nucleotide polymorphisms in the FABP4 gene may underlie the variance in such relationships.

## Subjects and Methods

### Subjects

The study was approved by the University of Louisville Human Research Committee, and informed consent was obtained from the legal caregiver of each participant. Consecutive children between the ages of 5 to 7 years attending public schools in Jefferson County were invited to participate in the study, after they underwent a school-based health screening, which included height and weight measurements. Based on such screening, children were identified when their BMI z score was ≥ 1.65 (OB) and age-, gender-, ethnicity-, and area-of residence-matched children with BMI z scores <1.65 (NOB) were then identified and recruited to serve as controls. Of note, all children were otherwise healthy, and were representative of the demographic characteristics of the general population of the city of Louisville http://ksdc.louisville.edu/sdc/census2000/cityprofiles/LouisvilleDP.pdf. Children were excluded if they had known diabetes or pre-diabetes http://www.diabetes.org/pre-diabetes/pre-diabetes-symptoms.jsp, any defined genetic abnormality or underlying systemic disease including hypertension, or if they were within a month from any acute infectious process.

### Anthropometry

To verify the school-health screening initial reports, children were weighed in a calibrated scale to the nearest 0.1 kg and height (to 0.1 cm) was measured with a stadiometer (Holtain, Crymych, UK). Body mass index (BMI) was calculated and BMI z-score was computed using CDC 2000 growth standards http://www.cdc.gov/growthcharts and online software http://www.cdc.gov/epiinfo. A BMI z score ≥ 1.65 was considered as fulfilling the criteria for obesity.

### Blood Based Assays

Blood samples were drawn by venipuncture in the morning after an overnight fast. Blood samples were immediately centrifuged and plasma was frozen at -80°C until assay. Plasma insulin levels were measured using a commercially available radioimmunoassay kit (Coat-A-Count Insulin; Diagnostic Products Inc). This method has a detection level of 1.2 μIU/mL and exhibits linear behavior up to 350 μIU/mL, with intra-assay and interassay coefficients of variability of 3.1% and 4.9%, respectively. Plasma glucose level was measured using a commercial kit based on the hexokinase-glucose-6-phosphate dehydrogenase method (Flex Reagent Cartridges; Dade Behring, Newark, DE). Insulin resistance was assessed using the homeostasis model assessment (HOMA) equation (fasting insulin × fasting glucose/22.5) [23].

Plasma hsCRP levels were measured within 2-3 hours after collection using the Flex reagent Cartridge (Date Behring, Newark, DE). This method has a detection level of 0.05 mg/dl, and exhibits linear behavior up to 255 mg/dl, with intra-assay and inter-assay coefficients of variability of 9% and 18% respectively for hsCRP. Serum lipids including total cholesterol, high-density lipoprotein (HDL) cholesterol, calculated low-density lipoprotein cholesterol (LDL), and triglycerides (TG) were also assessed using Flex Reagent Cartridges (Dade Behring).

Plasma FABP4 levels were also measured using commercial ELISA kits (ALPCO Diagnostics, Salem, NH) following the manufacturer's instructions. All assays were performed in duplicate and a calibration curve was included in each assay.

### DNA Extraction

Peripheral blood samples were collected in vacutainer tubes containing EDTA (Becton Dickinson, Franklin Lakes, NJ, USA). All DNA samples were extracted using QIAmp DNA blood kit (Qiagen, Valencia, CA) according the manufacturer's protocol. The concentration and quality of the DNA were determined using a ND-1000 Spectrophotometer (Nanodrop Technologies, Wilmington, DE, USA). The purity of the DNA were determined by calculating the ratio of absorbance at 260/280 nm, and all DNA samples had a ratio of 1.8-1.9. The precise length of genomic DNA was determined by gel electrophoresis using 1% agarose gel. All the purified samples were stored at -80°C until further analyses.

### Genotyping using Real-Time PCR

Genotyping was performed using the ABI PRISM 7500 Sequence Detection System for allelic discrimination following the manufacturer's instructions (Applied Biosystems). Four FABP4 single nucleotide polymorphisms, namely rs1051231, rs2303519, rs16909233 and rs1054135 were examined in this study (Applied Biosystems). All polymorphisms were genotyped using TaqMan technology (Applied Biosystems, Inc.). Two fluorogenic minor groove binder probes were used for each locus using the dyes 6-carboxyfluorescein (FAM; excitation, 494 nm) and VIC (excitation, 538 nm) which are easily differentiated in PCR system. Real-time PCR reaction was performed using 12.5 μl of TaqMan 2× universal master mix (Applied Biosystems, CA), 1.25 μl of SNP, 10.25 μl of RNase- and DNase-free water (Ambion, Austin, TX), and 1 μl of sample DNA, in a total volume of 25 μl per single well reaction. Two wells of a 96 well-plate (Applied Biosystems, CA) were used for each sample for each of the 4 single nucleotide polymorphisms. DNase-free water used as non-template control was included in each assay run. Assay conditions were 2 min at 50°C, 10 min at 95°C, and 40 cycles of 95°C for 15 s and 60°C for 1 min. Initially, the SNP assay was set up using SDS, version 2.1, software (Applied Biosystems, CA) as an absolute quantification assay, but after assay completion the plate was read using the allelic discrimination settings. Post-assay analysis was performed using the SDS software.

### Statistical Analysis

Data were expressed as mean ± SD. Significant differences within groups were analyzed using ANOVA for continuous variables and chi-square tests for categorical variables. Spearman's correlation analyses were conducted to examine potential associations between BMI and plasma concentrations of the various inflammatory mediators. Statistical analyses were performed using SPSS software (version 16.0; SPPS Inc., Chicago, Ill.). All p-values reported are 2-tailed with statistical significance set at <0.05.

## Results

A total of 182 OB children and 127 age-, gender- and ethnicity-matched NOB children were recruited during January to May 2008. The demographic characteristics of this cohort are shown in Table 1, and are virtually identical to the published demographics of the city of Louisville, Kentucky.

**Table 1:** Demographic characteristics and fasting morning plasma concentrations of lipids, glucose, insulin, hsCRP, and FABP4 in obese and matched non-obese children

	OB (n = 182)	NOB (n = 127)	P-value
**Age (years)**	6.7 ± 0.4	6.7 ± 0.4	
**Gender (% male)**	51.4	51.9	
**Ethnicity**			
**White Caucasian %**	75.7	76.6	
**African American %**	18.9	18.2	
**BMI z score**	2.04 ± 0.23	0.91 ± 0.12	<0.00001
**Cholesterol**	176.5 ± 3.1	164.4 ± 2.5	NS
**Triglycerides**	85.1 ± 4.1	70.4 ± 2.9	<0.01
**HDL**	49.6 ± 1.5	58.7 ± 1.0	<0.01
**VLDL**	18.9 ± 1.4	15.7 ± 0.9	<0.02
**LDL**	98.4 ± 2.4	87.4 ± 2.1	<0.002
**Glucose**	81.3 ± 1.3	78.2 ± 1.7	NS
**Insulin**	12.9 ± 1.3	6.5 ± 1.0	<0.001
**HOMA**	2.6 ± 0.3	1.4 ± 0.3	<0.001
**hsCRP ((mg/dl))**	2.67 ± 0.48	1.04 ± 0.13	<0.001
**FABP4 (ng/mL)**	21.4 ± 1.2	12.1 ± 1.0	<0.0001

As anticipated, OB children had higher HOMA values, indicative of insulin resistance, and also exhibited higher LDL, VLDL, and TG levels and lower HDL concentrations compared to NOB children (Table 1). OB children also had significantly higher levels of hsCRP and FABP4 than NOB (Table 1).

FABP4 levels were positively correlated with BMI z score (r:0.57; p < 0.0001). hsCRP and HOMA were also significantly associated with FABP4 (r = 0.29; p < 0.001 and r = 0.18; p < 0.01, respectively). However, FABP4 levels did not exhibit any linear correlation with lipid levels. Multivariate analyses of variance revealed that after adjusting for BMI z score, FABP4 contributed to 9% of the variance in hsCRP (p < 0.003) and 3% of the variance in HOMA (p < 0.05).

The frequency of each of the FABP4 polymorphisms is shown in Table 2 for OB and NOB children. Obese children showed increased frequency of rs1054135 polymorphism compared to control children. There were no differences between OB and NOB for the other 3 polymorphisms.

**Table 2:** Allelic distribution of FABP4 SNPs in obese and non-obese children.

SNP	Allele	OB (n = 182)	NOB (n = 127)	*p value*
**rs1054135**	GG	61 (33.5)	135 (74.2)	***<0.001***
	**GA**	**73 (40.1)**	**26 (14.3)**	
	**AA**	**48 (26.4)**	**19 (10.4)**	
**rs1051231**	AA	182 (100)	127 (100)	*N/A*
	AC	0	0	
	CC	0	0	
**rs2303519**	CC	116 (63.7)	100 (78.7)	*0.13*
	CT	65 (36.25)	26 (20.4)	
	TT	1 (0.05)	1 (0.9)	
**rs16909233**	GG	129 (70.8)	99 (77.9)	*0.26*
	AG	52 (28.6)	26 (20.5)	
	AA	1 (0.6)	2 (1.6)	

Only the rs1054135 polymorphism showed significant contributions to higher FABP4 plasma levels (Table 3). In contrast, obesity interacted with rs16909233 allelic variance, such that the presence of this single nucleotide polymorphism in the context of obesity was associated with higher HOMA values, indicative of insulin resistance (p < 0.001).

**Table 3:** HOMA and FABP4 plasma levels in variant and wild allele of SNPs in obese and non-obese children.

SNPs	OB (n = 182)		*p value*	NOB (n = 127)		*p value*
**rs1054135 G/A**	**A+ (n = 121)**	**A- (n = 61)**		**A+ (n = 35)**	**A- (n = 92)**	

FABP4 levels	22.5 ± 5.5	16.1 ± 2.4	*<0.001*	12.5 ± 3.5	7.7 ± 2.3	*<0.001*

HOMA	2.5 ± 0.7	2.6 ± 0.5	*>0.05*	1.5 ± 0.4	1.3 ± 0.4	*>0.05*

**rs1051231 A/C**	**C+ (n = 0)**	**C- (n = 186)**		**C+ (n = 0)**	**C- (n = 123)**	

FABP4 levels	N/A	19.2 ± 4.3	*N/A*	N/A	9.1 ± 3.4	*N/A*

**rs2303519 C/T**	**T+ (n = 66)**	**T- (n = 116)**		**T+ (n = 27)**	**T- (n = 100)**	

FABP4 levels	19.7 ± 5.4	19.7 ± 5.3	*>0.05*	11.3 ± 3.2	10.2 ± 3.1	*>0.05*

HOMA	2.5 ± 1.0	2.6 ± 0.5	*>0.05*	1.4 ± 0.4	1.4 ± 0.4	*>0.05*

**rs16909233 G/A**	**A+ (n = 53)**	**A- (n = 129)**		**A+ (n = 28)**	**A- (n = 99)**	

FABP4 levels	19.2 ± 5.6	19.2 ± 5.2	*>0.05*	11.0 ± 2.7	10.8 ± 3.3	*>0.05*

HOMA	3.1 ± 1.1	2.2 ± 0.9	*<0.001*	1.5 ± 0.4	1.4 ± 0.4	*>0.05*

## Discussion

In this study, we show that FABP4 plasma levels are significantly higher in obese children and correlate with measures of insulin resistance and systemic inflammation, such as HOMA and hsCRP, respectively. Furthermore, the frequency of the rs1054135 single nucleotide polymorphism in the FABP4 gene was higher in OB compared to NOB, and seemingly contributes to higher FABP4 levels, while the presence of rs16909233 is associated with insulin resistance in the context of obesity.

Before we discuss the potential implications of these findings, some methodological issues deserve comment. This was a community study that was designed such as to be as representative as possible of the ethnic distribution of the population of Louisville, and a priori excluded any child with known diabetes, hypertension or another chronic disease condition associated with obesity. Such methodological approach may have artificially reduced the impact of obesity on FABP4 levels as well as the magnitude of the association of any given FABP4 allelic variant with the degree of metabolic dysfunction or FABP4 plasma levels. Therefore, it will be important to explore such possibilities in a clinical referral obese pediatric population. Secondly, we restricted the age range of our cohort to a narrow time window that is associated with the initial 3 years of attendance within the public school system, a period that coincides with significant changes in food consumption patterns [24,25]. Such decision aimed to reduce as much as possible the presence of confounders across a wide age spectrum. Finally, we identified and recruited closely matching control children in the same school for each obese child that was recruited, such as to nullify as best as possible some of the known potential confounders that could be introduced in the process of cohort selection.

FABP4 has been proposed as a bridge between inflammatory processes and other biological pathways related to the metabolic syndrome. Our current findings are in close agreement with the only 2 other published studies in children, in which obesity was a risk factor for elevated plasma FABP4 levels [21,22]. However, Reinehr et al reported significant correlations between FABP4 and percentage body fat and leptin but the absence of any significant association between FABP and any of the markers of the metabolic syndrome [21]. In a more recent study from Korea, FABP4 concentrations were significantly correlated with BMI and waist circumference, but failed to exhibit a significant association with insulin sensitivity after adjusting for BMI [22]. The larger cohort size in the present study, allowed not only for confirmation of the strong association between FABP concentration and BMI, but also indicated the presence of weaker, albeit significant associations with insulin sensitivity and systemic inflammation.

To further examine the variance in FABP4 levels across obese and non-obese children, we initially examined the frequency of several FABP4 gene polymorphisms in our cohort. This approach revealed that the rs1054135 polymorphism was significantly more prevalent among obese children (Table 2), while no such differences were apparent among the 3 other polymorphisms tested. We are only aware of another published study on FABP4 genomic variants in humans. Indeed, in a population-based genetic study that included 7,899 participants, individuals that carried a T-87C polymorphism in the FABP4 gene, which reduces the transcriptional efficiency of the FABP4 gene, had lower serum triglyceride levels and significantly reduced risk for coronary heart disease and type 2 diabetes compared with subjects homozygous for the wild-type allele [26]. Other SNP were not specifically examined, such that comparisons across this study and the present study are not possible. We should emphasize however, that FABP4 is also expressed in activated macrophages [27-29], and high FABP4 concentrations are detected in human atherosclerotic lesions [30]. Thus, alleles that reduce FABP4 expression or activity should be metabolically protective, while those associated with higher FABP4 may increase susceptibility. In this regard, genetic variability at the FABP4 as evidenced by rs1054135, increased FABP4 plasma levels in both obese and non-obese children, and its relative preponderance in obese children suggests an interaction between obesity and FABP4 alleles to increase FABP4 circulating levels. Furthermore, the rs16909233 allelic variants were not differentially present in OB and NOB children, and did not alter plasma FABP4 levels. However, the presence of this allelic variant seemed to modify insulin resistance in the context of obesity. Taken together, these initial findings in a pediatric community based cohort, suggest that FABP4 genomic variance may be an important determinant for cardiometabolic risk, particularly among obese children.

In summary, we have shown that young obese school-aged children exhibit higher circulating levels of the pro-atherogenic and pro-inflammatory FABP4, which in turn, may contribute to the risk for reduced insulin sensitivity and increased systemic inflammation, as illustrated by elevated hsCRP levels. Furthermore, gene variants in FABP4 appear to differentially contribute to the pro-inflammatory or diabetogenic potential of obesity during childhood.

## List of Abbreviations

BMI: body mass index; FABP4: fatty acid binding protein 4; HOMA: homeostasis model assessment; hsCRP: high sensitivity C reactive protein; NOB: non-obese; OB: obese; SNPs: single nucleotide polymorphisms.

## Competing interests

The authors declare that they have no competing interests.

## Authors' contributions

AK, BB, MH, JK, LKG, RB, OSC collected data, conducted assays, performed data analyses, and contributed to manuscript editing. LKG, RB, OSC and DG recruited subjects. DG initiated the project, reviewed data analysis and drafted the initial manuscript. All authors have reviewed and approved the final manuscript.
